# Dexmedetomidine premedication increases preoperative sedation and inhibits stress induced by tracheal intubation in adult: a prospective randomized double-blind clinical study

**DOI:** 10.1186/s12871-022-01930-z

**Published:** 2022-12-21

**Authors:** Jun Xiong, Jie Gao, Yanan Pang, Yafen Zhou, Yongxing Sun, Yanyan Sun

**Affiliations:** 1 Department of Anesthesiology, Shenzhen University General Hospital, Shenzhen University, Xueyuan AVE 1098, Nanshan District, Shenzhen, Guangdong 518055 China; 2 Department of Anesthesiology, First Affiliated Hospital of Kunming Medical University, No. 295 Xichang Road, Kunming, Yunnan 650032 China; 3 Department of Anesthesiology, Sanbo Brain Hospital, Capital Medical University, No. 50 Yikesong, Xiangshan, Haidian District, Beijing, 100093 China

**Keywords:** Dexmedetomidine, Premedication, Sedation, Stress, Anxiety, Tracheal intubation

## Abstract

**Objective:**

The aim of this prospective randomized double-blind study is to evaluate whether oral dexmedetomidine (DEX) premedication could increase sedation in order to reduce preoperative anxiety and inhibit stress response during general anesthesia tracheal intubation.

**Materials:**

A total of 100 ASA I and II adult patients undergoing elective neurosurgery were randomly divided into the control group (C group, *n* = 50) and the oral DEX premedication (DEX group, *n* = 50). Patients were administrated 4 μg/kg dexmedetomidine orally pre-anesthesia 120 min. Hemodynamic variables were monitored and recorded from premedication to 10 min after tracheal intubation. The primary outcome, the sedation level of all participants, was evaluated by Richmond Agitation Sedation Scale (RASS), and Numerical Rating Scale was to measure their intensity of thirst and satisfaction of patients’ family members. During general anesthesia induction, the total dosage of induction anesthetics and complications relative to anesthesia induction were recorded. After tracheal intubation, blood sample was drain from radial atrial line to measure levels of adrenocorticotropic hormone (ACTH) and cortisol.

**Results:**

RASS scores at 60 min after premedication and on arrival in the operating room were significantly reduced in the DEX group (*P* < 0.001). Oral DEX premedication not only increased the intensity of thirst but also the satisfaction of their family members (*P* < 0.001). The cortisol level after tracheal intubation was deduced by oral DEX premedication (*P* < 0.05). Oral DEX premedication reduced heart rate (HR) and mean arterial pressure (MAP) on arrival in the operating room, and HR when tracheal intubation (*P* < 0.05). During the whole process of anesthesia induction, although the lowest MAP in two groups were not significantly different, the lowest HR was significantly lower in the DEX group (*P* < 0.05). Oral DEX premedication might reduce HR from premedication to 10 min after tracheal intubation. However MAP was reduced just from premedication to on arrival in the operating room. Total induction dosages of propofol, midazolam, sulfentanil and rocuronium were similar in two groups (*P* > 0.05), as well as the complications relative to anesthesia induction and cases of rescue dopamine therapy were similar (*P* > 0.05).

**Conclusion:**

Oral DEX 4 μg/kg premedication was an efficient intervention to increase preoperative sedation and reduce stress reaction induced by general anesthesia tracheal intubation, but also it was with the stable hemodynamic during the process of general anesthesia tracheal intubation, and improved the satisfaction of patients’ family members. In this study, the sparing-anesthetic effect of 4 μg/kg DEX oral premedication was not significant, and this would be needed to study in future.

**Trial registration:**

This trail was registered at Chinese Clinical Trial Registry (https://www.chictr.org.cn, Jie Gao) on 15/04/2021, registration number was ChiCTR2100045458.

## Introduction

The preoperative time is expected to be a stressful period for the majority of patients and their family members. Preoperative or pre-anesthesia anxiety is a huge impact on patients’ emotion and psychiatry, which produces physical problems correlated with autonomic fluctuation and incidence of nausea and vomiting, as well as postoperative pain. This stress also affects potentially anesthesia, for example increasing requirement of anesthetic [1]. If not be relieved, it could be detrimental to postoperative outcomes. Therefore, it stands to reason that adequate relief of preoperative anxiety translates into better perioperative outcomes [2].

To manage preoperative anxiety with premedication might demonstrate some effects against peri-operative anxiety, pain and other discomfort for surgeon and anesthesiologist to improve quality of anesthesia and surgical recovery [3]. Various medicines have been used preemptively to ameliorate preoperative discomfort and reduce side-effects of anesthetic [4], including midazolam, opioids, atropine, and so on. Opioids are the most usually used premedication [5], however which have to do with respiratory depression, nausea, and urinary retention. Midazolam, a benzodiazepine anxiolytic, is common for patients to experience preoperative anxiety because adequate sedation might ameliorate anxiety [6], which with not only rapid onset of action and a minimum hemodynamic effect, but also muscle relaxant properties [7]. However the most adverse effects secondary to midazolam include respiratory depression and postoperative cognitive changes [8]. Additionally, although sedative premedication is widely used to improve patients’ own experience, there is little clinical evidence to support sedative premedication or routine sedative premedication was demonstrated to be benefit in general anesthesia [9]. Hence consistent relieve of preoperative anxiety is still a major challenge, and there is a need for an ideal method which provides better preoperative stress relief with minimal side effects [10].

Dexmedetomidine (DEX) is a highly selective agonist activating pre- and postsynaptic α2 adrenoceptor. Because of a significantly greater α2:α1 adrenoceptor affinity ratio, DEX demonstrates both pharmacokinetic and pharmacodynamics advantages without directed γ-aminobutyric acid (GABA)-ergic effect [11]. With sedative, anxiolytic, and analgesic features, it is a useful adjuvant to the anesthesia protocol. Additionally, it has been shown anesthetic-sparing and conscious sedation effects without causing deleterious respiratory depression, these properties make it superior to other agents in perioperative setting, especially in the elderly and pediatric population [12]. Thus it has been extensively studied in pediatric patients recently for premedication [13]. Although with various routes of administration, DEX is most usually used as continuous infusion, now it has been increasingly studied as an alternative to standard medicine for oral and intranasal administration [14, 15]. Compared to intravenous and intranasal administration, oral route is noninvasive and easier to administer, meanwhile it has better medication acceptance for patients, particular for pediatric patients [16].

However, there has been lack of studies on oral DEX premedication in adult patients. The aim of this prospective randomized double-blind study is to evaluate whether oral DEX premedication could increase sedation in order to reduce preoperative anxiety and inhibit stress response during general anesthesia tracheal intubation.

## Materials and methods

### Study

This prospective randomized double-blind clinical study protocol was registered prior to patients recruitment at Chinese Clinical Trail Registry (https://www.chictr.org.cn, Jie Gao) on 15/04/2021, the registration number was ChiCTR2100045458. And the present study was approved by the ethics committee of our hospital (SBNK-YJ-2021-008-02). All participants participated in the study signed the written informed consent. All methods were performed according to the guidelines in Ethics Approval and the consent. The study was conducted in accordance with the Consolidated Standards of Reporting Trials (CONSORT) statement and the Declaration of Helsinki.

### Patients

Patients scheduled for elective neurosurgery under general anesthesia were recruited at this tertiary hospital from May to July in 2021. A total of 100 participants between 18 years and 70 years with American Society of Anesthesiologists physical status I and II were enrolled. Detailed exclusion criteria were as follows: age < 18 or > 70 years, abnormal responses to DEX treatment (transient hypertension, nausea induced by DEX), severe organ dysfunction (for example, hepatic, kidney dysfunction, and so on), sinus bradycardia, pituitary tumor, severe obstructive sleep apnea, morbid obesity (body mass index ≥35 kg/m^2^), allergy to DEX, systolic blood pressure < 90 mmHg and heart systolic dysfunction, hormone disorder and/or hormone treatment, adrenocortical dysfunction, multiple operations, and disturbance of consciousness.

### Randomization and masking

**Fig. 1:**
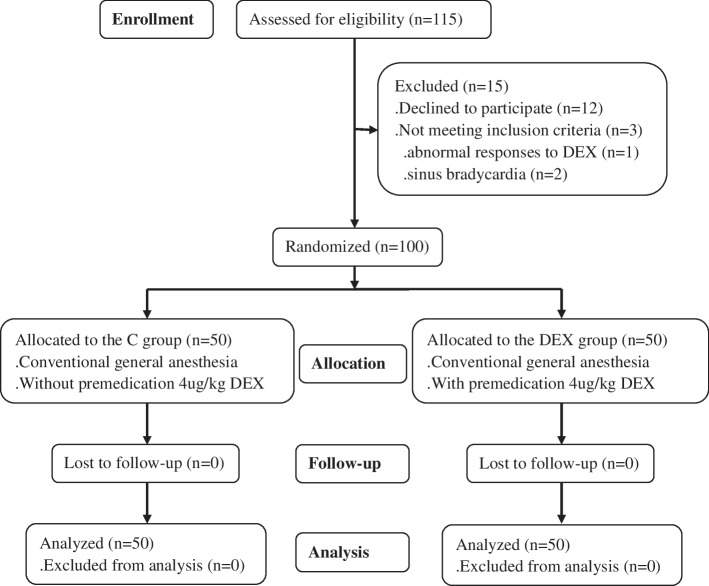
CONSORT flow diagram of this study

All eligible patients were randomly divided in a 1:1 ratio into two groups using a SPSS random number generator producing randomization sequence, the control group (C group) and the oral DEX premedication group (DEX group) (Fig. 1). The randomization was done by an anesthesiologist without further involvement in this study. Allocation concealment was implemented with sequentially-numbered sealed envelopes. All participants and investigators were blinded to the treatment allocation. Unblinding was done after the end of the four weeks intervention period.

### Study protocol

All participants in the DEX group, 4 μg/kg DEX (No. 1911251, Guorui Pharmaceutical Co., Ltd., Leshan, Sichuan, China.) were administered orally 120 min before general anesthesia induction, and oral medicine was diluted to a final volume of 10 ml with normal saline. The same volume normal saline was administered orally to all patients in the C group at the same time interval. An independent anesthesiologist performed participant random assignment and prepared premedication medicine.

Heart rate (HR), pulse oxygen saturation (SpO_2_%), noninvasive blood pressure (NIBP) of all patients were monitored continuously and recorded automatically at 15 min interval till to 120 min when transferred to the operating room. Richmond Agitation-Sedation Scale (RASS) scores were recorded before DEX administration and 60 min after administration to measure sedation level. The premedication treatment was implemented by another anesthesiologist who was blinded to medicines.

On arrival in the operating room, standard physiological signs monitoring were still performed continuously, such as electrocardiogram (ECG), SpO2%, NIBP, and HR. Before anesthesia induction, sedation status of patients was evaluated again, at the same time their thirst level was also measured with Numerical Rating Scale (NRS) (range 1–10, the higher score, the more intensity of thirst) [17]. Intravenous access was secured with 18G cannula and lactated ringer’s solution was infused in all participants, who received preoxygenation with 100% O_2_ via a facial mask for 5 min. General anesthesia was induced with administration of propofol, midazolam, sulfentanil, and relaxed with rocuronium according on patients’ body weight and anesthetist’s personal experience. Bispectral Index (BIS, Aspect Medical System, Natick, MA, USA) was maintained between 45 ~ 60. When hypoventilation appeared and loss of consciousness, assisted ventilation was initiated. After confirming muscle relaxation, tracheal intubation was performed successfully once by a senior anesthesiologist with video laryngoscope.

Throughout the induction of anesthesia, the standard monitoring were utilized to pay close attention to vital signs, including systolic blood pressure (SBP), diastolic blood pressure (DBP), mean arterial pressure (MAP), HR, SpO_2_%, ECG, exhaled carbon dioxide concentration (EtCO_2_). The investigator recorded hemodynamic variables from DEX premedication to 10 min after tracheal intubation, that was, premedication time as the basal level (T0), 15, 30, 45, 60 min after premedication (T1, T2, T3, T4), before starting anesthesia induction (T5), 1, 2, 3, 5 min after anesthesia induction (T6, T7, T8, T9), immediately after intubation (T10) and 10 min after intubation (T11), respectively. Except hemodynamic parameters, the total dosages of anesthetics, the lowest MAP and HR in the process of anesthesia induction were recorded too. If HR was lower than 50 bpm constantly during induction stage, atropine 0.005 mg/Kg was administered intravenously. Induction hypotension was defined according to MAP< 60 mmHg [18], dopamine was administrated to remedy hypotension if it sustained.

After tracheal intubation, patients underwent radial arterial cannulation for continuous arterial pressure monitoring and drawing blood samples of adrenocorticotropic hormone (ACTH) and cortisol. The radial arterial cannulation was finished by the same senior anesthesiologist who completed tracheal intubation. The independent anesthetist, who responsible for general anesthesia induction and maintain, was blinded to patients’ assignment. After patients arriving in the operating room, the satisfaction degree of their family member who accompanied with the patient was evaluated with NRS by an independent anesthetist.

### Outcomes

The primary outcome of this study was the difference of patients’ sedation, which was based on RASS score before DEX premedication, at 60 min after DEX premedication and on arrival in the operating room. The secondary outcomes were the intensity of stress response induced by tracheal intubation, as well as hemodynamic variables during general anesthesia induction and tracheal intubation, and the total dosages of induction anesthetics. The degree of satisfaction of patients’ family member was also included into the secondary outcomes.

### Sample size and statistics analysis

Based on our pilot study, we found, mean (SD) of RASS score were − 0.2 (0.748) in the DEX group (*n* = 5) and 0.2 (0.4) in the C group (*n* = 5), respectively. The sample size was calculated with power of 0.8 and alpha error of 0.05. At least 37 patients per group were needed. Assuming a 10% dropout rate and increasing credibility of this study, 100 patients were enrolled (50 subjects for each group).

Statistical analysis was performed through IBM SPSS version 21 for Windows (IBM SPSS Inc., Beijing, China). Continuous variables are expressed as mean ± SD, and categorical variables were presented as number (percentage). Kolmogorov-Smirnov test was used to evaluate normality of continuous variables, Levene’s test was for equality of variances, and their differences were analyzed via independent *t* test or Mann-Whitney *U* test where appropriate. Categorical variables were evaluated via Pearson *χ*^2^ test or Fisher exact test, where appropriate. The differences of hemodynamic variables in two groups were analyzed with repeated measured analysis, and the variables between two groups at each time point were compared via independent *t* test or Mann-Whitney *U* test. *P* < 0.05 was considered as significant.

## Results

### General data

A total of 115 patients were recruited, and 100 patients completed this study. There were no statistically significant differences between two groups with respect to demographic characteristics, including age, height, weight and body mass index (*P* > 0.05). All patients recruited in this study were ASA I and II class without severe organ dysfunction, such as hepatic, kidney and heart dysfunction, and so on. All of them underwent craniotomy operation without obvious high intracranial pressure on eight-clock on morning. The C group included 25 (50%) female and 25 (50%) male patients, and 27 (54%) female and 23 (46%) male patients in the DEX group. There were one case of diabetes and 4 cases of hypertension in the C group, and one case of diabetes and 6 cases of hypertension in the DEX group, their conditions were controlled strictly. Except medications controlling blood glucose and hypertension, there was no special treatment for all patients. Postoperative pathological examination demonstrated there were vestibular schwannoma 31 (31%), meningioma 27 (27%), cholesteatoma 8 (8%) and glioma 34 (34%).

### The degree of sedation, thirst and satisfaction of family member, levels of cortisol and ACTH, and dosage of anesthetics in two groups

**Fig. 2:**
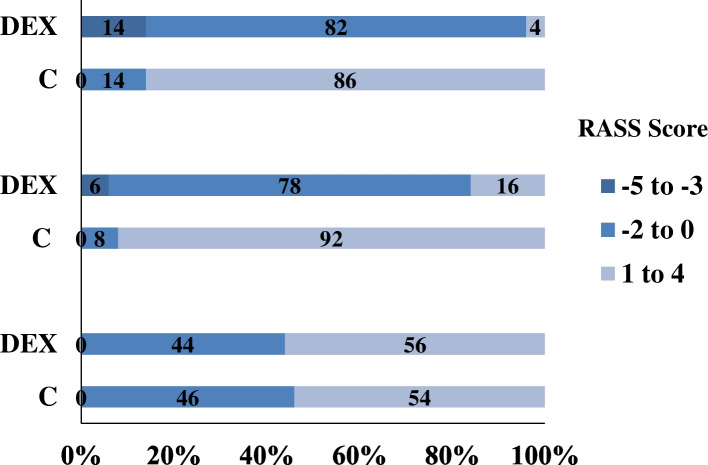
Distribution of RASS scores before DEX premedication, 60 min after DEX premedication and arrival in the operating room. C: the Control group, DEX: the DEX group

The RASS sedation score before DEX premedication, at 60 min after premedication and on arrival in the operating room were compared between two groups, DEX significantly increased level of patients’ sedation (Table 1) and proportion of patients’ sedation (Fig. 2). Meanwhile, the degree of patients’ thirst and satisfaction of their family members were compared too (Table 2). Both thirst NRS scores and satisfaction NRS scores in the DEX group were significantly higher than these in the C group (*P* < 0.05). The value of cortisol was significantly reduced in the DEX group (*P* < 0.05), but there was no statistical difference of ACTH between two groups (*P* > 0.05). The total dosages of anesthetics during general anesthesia induction were demonstrated in the Table 2. No significant statistical difference was shown in two groups.

**Table 1:** The Richmond Agitation Sedation Scale (RASS) in two groups

	C (*n* = 50)	DEX (*n* = 50)	*P*
Before DEX premedication	0.7 ± 0.8	0.7 ± 0.8	0.775
60 min after DEX premedication	1.24 ± 0.66	−0.78 ± 1.17	0.000
arrival in the operating room	1.12 ± 0.63	−0.90 ± 1.11	0.000

*C* The Control group, *DEX* The DEX group

**Table 2:** NRS scores, satisfaction of family member, levels of cortical and ACTH, and the dosages of anesthetics in two groups

	C (*n* = 50)	DEX (*n* = 50)	*P*
NRS of thirst	2.2 ± 1.4	3.7 ± 2.4	0.001
NRS of satisfaction of family member	1.2 ± 0.7	3.6 ± 1.5	0.000
ACTH (pg/ml)	15.1 ± 11.8	12.1 ± 9.7	0.344
Cortisol (ug/ml)	11.5 ± 10.4	8.5 ± 4.7	0.033
Sulfentanil (ug)	25.5 ± 8.6	24.6 ± 7.0	0.983
Propofol (mg)	102.3 ± 29.6	100.4 ± 29.2	0.754
Midazolam (mg)	2.9 ± 1.3	2.6 ± 1.5	0.513
Rocuronium (mg)	78.8 ± 19.1	73.2 ± 18.6	0.151

*C* The Control group, *DEX* The DEX group, *NRS* Numerical Rating Scale

### The incidence of side-effects and hemodynamic variety

There was no difference with respect to the incidences of induction hypotension and bradycardia in two groups. It was same related to cases of rescue dopamine (Table 3).

**Table 3:** The incidences of induction hypotesion, bradycardia and cases of rescue dopamine in two groups

	Induction Hypotesion^a^	Bradycardia^b^	Cases of Rescue Dopamine
C (*n* = 50)	3 (6%)	5 (10%)	5 (10%)
DEX (*n* = 50)	3 (6%)	5 (10%)	6 (12%)
*χ*^*2*^	0.000	0.000	0.102
*P*	1.000	1.000	0.749

*C* The Control group, *DEX* The DEX group

^a^Induction hypotesion: MAP less than 60 mmHg

^b^Bradycardia: HR less than 50 bpm

The hemodynamic parameters at key time points from DEX premedication to 10 min after tracheal intubation was displayed in the Table 4, including the basal level, on arrival in the operating room, tracheal intubation, and so on. At these key time points, DEX premedication significantly reduced HR but not MAP (*P* < 0.05). Besides on arrival in the operating room, MAPs were similar in two groups at other key time points (*P* > 0.05).

**Table 4:** The hemodynamics at key time points in two groups

	**C (*n* = 50)**	**DEX (*n* = 50)**	***t***	***P***
MAP (mmHg)				
basal	96.1 ± 11.0	92.4 ± 12.1	1.589	0.115
arrival in operating room	101.7 ± 10.9	93.2 ± 13.5	3.436	0.001
intubation	97.1 ± 15.5	96.6 ± 17.3	0.176	0.860
10 min after intubation	86.3 ± 13.5	90.3 ± 14.8	−1.431	0.156
the lowest	78.1 ± 10.9	82.6 ± 15.0	−1.723	0.088
HR (bpm)				
basal	78.3 ± 8.2	78.4 ± 7.2	−0.065	0.948
arrival in operating room	76.0 ± 14.4	65.8 ± 11.5	3.945	0.000
intubation	82.5 ± 16.2	72.0 ± 13.9	3.481	0.001
10 min after intubation	76.0 ± 17.6	66.7 ± 11.6	3.132	0.002
the lowest	66.0 ± 12.4	59.7 ± 9.0	2.908	0.005

*C* The Control group, *DEX* The DEX group

**Fig. 3:**
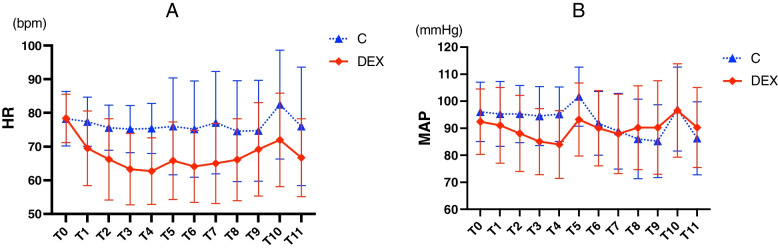
The general trend of HR (**A**) and MAP (**B**) from DEX premedication to 10 min after tracheal intubation. Bpm: beats of per minute. C: the Control group, DEX: the DEX group

The general trend of hemodynamic, including MAP and HR from DEX premedication to 10 min after tracheal intubation, was analyzed with repeated measures (Fig. 3). DEX reduced participants’ HR rather than MAP during the whole study (Fig. 3A). Actually, before onset of anesthesia induction, DEX premedication also reduced patients’ MAP (Fig. 3B).

## Discussion

This prospective, double-blind, randomized controlled study showed that oral DEX 4 μg/kg premedication could increase patients’ preoperative sedation and inhibit the stress reaction induced by general anesthesia tracheal intubation, because of reduction of hemodynamic variables and the level of cortisol. This premedication also brought other advantages without obvious side-effects, including reduction of salivary and higher satisfaction of patients’ family member.

Most patients undergoing elective surgery and anesthesia experience preoperative or pre-anesthesia anxiety, even they are afraid of surgery and anesthesia [19]. The serious anxiety might bring negative impact on psychological and somatic, and in consequence affect anesthesia, surgery and postoperative therapy and rehabilitation. Therefore reducing intensity of preoperative anxiety might improve patients’ prognosis [20]. Recently, DEX boasted an increasing use in surgical premedication, as an alternative of medizolam, however this method is limited in pediatric patients via intranasal premedication [21], and there is lack of study about intranasal or oral DEX in adult. Our result of lower RASS score in the DEX group also demonstrated that DEX premedication could increase pre-anesthesia sedation and ameliorate anxiety in adult patients.

In our study, DEX premedication was administrated orally because oral route is a nontraumatic route and more acceptable than intravenous route [22]. Additional, the total volume of oral premedication was 10 ml and administrated 120 min before anesthesia induction, so this implement complied strictly with ASA fasting guideline. Meanwhile we considered that this implementation might be able to decrease discomfort caused by fasting. In the study of Kundra, patients performed similar sedation and hemodynamic trend as these in our study with oral administration DEX 4 μg/kg. Moreover, DEX could produce quite pain relief and less salivation [23]. So DEX was considered as more suitable premedication agent for patients undergoing general anesthesia because of its effects of sedation, analgesia and anti-salivation. All of these effects have to be provided via combination of benzodiazepine, opioids and anti-cholinergic agents in conventional clinical practice. In other words, DEX might replace these three sorts of agents as premedication. Even oral DEX might achieve good preoperative sedation level in uncooperative patient, whose Ramsey score was 4 ~ 5 and similar as sedation level in our study [24].

In the present study, oral DEX premedication increased intensity of sedation significantly with less anxiety, but also DEX premedication decreased patients’ MAP and HR till on arrival in the operating room. Undoubtedly, this protective effect might improve preoperative and pre-anesthesia patients’ comfort, and there was no obvious side-effects with respected to 4 μg/kg DEX oral. Recently, s meta-analysis showed that DEX premedication could reduce HR about 16.9 bpm and MAP about 12.8 mmHg during tracheal intubation [25]. In our study, DEX premedication also showed HR and MAP reduction when intubation, especially HR was reduced 10.5 bpm. This result was consisted with that of meta-analysis. DEX has been widely used in general anesthesia, and its hemodynamic abnormalities, particularly bradycardia should be weighted [26]. However in this study, there was no obvious bradycardia and hypotension needed vasoactive agent rescue therapy before anesthesia induction, this result was consisted with the previous studies [22, 23]. Even if there were hemodynamic unstable and rescue dopamine usage during anesthesia induction, they were similar in two groups. Based on the previous study, on matter to adult and pediatric patients, 4 μg/kg DEX oral was a safe dosage with suitable sedation and less side effects due to relative lower bioavailability of oral DEX [22, 23]. Hence oral 4 μg/kg DEX was adopted in our study.

In our study, oral DEX premedication also inhibited stress response caused by general anesthesia tracheal intubation, because this protocol performed lower cortisol level compared with the control group. Blood cortisol level has been the most widely used as a biomarker of stress response [27]. During tracheal intubation the level of cortisol was significantly increased due to this strong stress response [28], therefore oral DEX premedication might inhibit this stress. These results were consisted with the previous study, where DEX was administrated intravenously preoperatively [29]. During acute stress, ACTH level rises initially in a pattern of large surge followed by cortisol increasing. With prolongation of stress response, ACTH would return to basal levels [30]. This means that cortisol level rising lags ACTH about 5 ~ 20 min. Its peak blood value achieves in 10 ~ 30 min after stress [27]. In our study, because blood sample was drained from radial atrial line built after tracheal intubation. This time delay might explain the reason of DEX premedication reducing cortisol level instead of ACTH level. Another explanation for similar ACTH levels in two groups might be much lower dosage of DEX in our study. In humans, wakeable sedation was required between 0.2 ~ 0.3 ng/ml plasma concentration, thus the least dosage of 300μg DEX was needed via oral route [31]. The mean body weight of our study was less than 70 kg, so the mean dosage of DEX was less than 300μg.

The perioperative sparing-anesthetic effect of DEX has been well known in many surgical settings via various administrating routs [32, 33]. In our study, the dosages of propofol, midazolam, sulfentanil and rocuronium were not different in two groups. Additional the level of BIS and MAP at time intervals of tracheal intubation and 10 min after intubation was similar in two groups. These results did not demonstrated reductions in anesthetics and opioid requirements with oral DEX premedication. The low DEX dosage was speculated to produce the distinction from the previous studies.

The function of salivary glands is strictly controlled by autonomic nervous system. Stress response might cause dramatic changes in salivary glands secretion [34]. The previous studies showed that DEX reduced salivary secretion, which were consisted with our result, because patients with DEX premedication felt more thirst compared to the control group. We speculated the anti-sialogogue effect of DEX increased patients’ feeling of thirst. This effect is beneficial to prevent choking on saliva during anesthesia induction [35].

Another major novel finding of the present study was oral DEX premedication might improve preoperative satisfaction of patients’ family members who accompanying with these patients. Although family members of patients undergoing surgery could experience serious anxiety, there is lack of study with respect to perioperative anxiety and satisfaction of patients’ family [36]. In fact we considered the higher satisfaction from patients’ family members could encourage them to give more support to these patients. These supports from the dedicated family impact patients’ recovery [37].

### Limitation

In our study there were still certain limitations to be considered when reviewing the results. Firstly, the anxiety level was not included. Because although there are many scales measuring preoperative anxiety (for example STAI, HADS and APAIS), they are not enough simple to evaluate patients’ anxiety level. When these assessments had to be done after the patients were waked up from sedative status, we worried about these complicated questionnaire might increase patients’ anxiety and reduce their authenticity. Thus we had to adopt RASS to assess sedation level in order to reflect the patients’ status of anxiety. Additional, adequate sedation might ameliorate anxiety and emotional trauma [38], this was another reason why we used RASS to show the status of anxiety. In the future study, professional preoperative assessment of anxiety should be added to increase the credibility of results. Secondly, there is lack of better scale for the accuracy of thirst and long-term follow-up in this study. Thirdly, only neurosurgical patients were chosen for this study. Because, in our culture, craniotomy is considered as high-risk surgery, so it may increase the level of preoperative anxiety and difference between interventions. All of these would be considered in our future study, for example, intensive care unit length-of-stay, observation of long-term prognosis, the patient’s satisfaction with the sedation and the patients’ cognitive change. If possible, multicenter study will be hold including other diseases beyond neurosurgery. Additionally, placebo and other medicines would be adopted as the control groups in our future study.

## Conclusion

Oral DEX 4 μg/kg premedication was an efficient intervention to increase preoperative sedation, thus ameliorate anxiety for patients undergoing surgery and improve their family members’ satisfaction. This premedication was able to inhibit the stress reaction induced by general anesthesia tracheal intubation with the stable hemodynamic change and lower level of cortisol. At this dosage, the sparing-anesthetic effect of DEX was not significant, which should be studied in the further research.

## Data Availability

The datasets generated and/or analysed during the current study are available from the corresponding author by the email address b2008194@126.com.

## Abbreviations

DEX: Dexmedetomidine
NRS: Numerical Rating Scale
RASS: Richmond Agitation Sedation Scale
